# Using meta‐ethnography to develop a conceptual model of peer‐assisted learning of nursing students in clinical practice

**DOI:** 10.1002/nop2.229

**Published:** 2019-01-04

**Authors:** Matthew C. Carey, Bridie Kent, Jos M. Latour

**Affiliations:** ^1^ School of Nursing and Midwifery University of Plymouth Plymouth UK

**Keywords:** clinical practice, nurse education, peer‐assisted learning, undergraduate nursing

## Abstract

**Aim:**

The study presents the findings of a meta‐ethnographic study, developing a conceptual model for peer‐assisted learning for undergraduate nurses in clinical practice.

**Design:**

Qualitative meta‐ethnography.

**Methods:**

Meta‐ethnography was used to synthesize the findings of two ethnographic studies and a qualitative review related to the influence of peer‐assisted learning on student nurses in clinical practice.

**Results:**

Four key themes were identified underpinned by six sub‐themes: (a) “Social” whereby “connecting with peers” is an important part in peer‐assisted learning. (b) “Enabling” peers through “collaborative support for advice and guidance” and “reducing anxiety/increasing confidence.” (c) “Organizational” aspects in peer‐assisted learning in “establishing structure and navigating practice” and “establishing the role of the PAL.” (d) “Learning” as a product of developing knowledge and skills through “sharing of practice experience” and “enhancing knowledge of care.” The conceptual model presents a structure outlining elements required for developing effective knowledge and skills through peer‐assisted learning.

## 1 INTRODUCTION

Peer‐assisted learning (PAL) is an initiative whereby students acquire knowledge and skills through the support provided by matched or status equals (Topping, 2005). The exploration of PAL amongst nursing students in clinical settings by Carey, Chick, Kent, and Latour (2018) determined the consideration of matched equals to include student nurses across all years in a programme of study. Evidence of the use of PAL in nursing has been captured in both theoretical and simulated environments (Dumas, Hollerbach, Stuart, & Duffy, 2015; Williams & Reddy, 2016). However, the exploration of PAL in clinical practice settings is limited (Carey, Kent, & Latour, 2016). Furthermore, the advancement of PAL in nurse education has been hindered by its lack of definition and consistency of structure (Carey, Chick, et al., 2018; Secomb, 2008).

### 1.1 Background

In a recent systematic review, Carey, Kent, and Latour (2018) explored the experiences of nursing students engaging in PAL in clinical practice. They determined that student participation in PAL existed in both formal and informal circumstances. Synthesis of the most recent evidence supported the belief that engagement in PAL is beneficial, in terms of shared experience and learning, for developing clinical knowledge and skills. Furthermore, PAL helps in the forming of community, thereby reducing anxiety and increasing student confidence (Carey, Kent, et al., 2018). Recommendations from the review encouraged further exploration of the experience and influence of PAL amongst student nurses in clinical practice. A recent study exploring PAL in clinical settings found that there were further benefits arising from the shared learning experience and through navigating clinical practice (Carey, Chick, et al., 2018). The authors concluded that nursing students had much to gain from one another when engaging in PAL and therefore it was important to determine the current structures in place to support how PAL is implemented in clinical practice.

The evidence indicates that models for supporting student learning through PAL are limited. Christiansen and Bell (2010), in their exploration of peer learning partnerships, referred to a learning initiative developed in a university in the UK, but offered no depth of discussion in relation to structure and functionality. Furthermore, the emphasis in the results of the study was on developing relationships, noting factors such as emotional support, gaining acceptance and contributions towards supportive learning relationships (Christiansen & Bell, 2010). This was a feature presented by Roberts (2008, 2009), who highlighted that relationship and socialization practices were identified as potential keys to forming communities for the success of peer learning in practice.

A more recent model in development is the Collaborative Learning in Practice (CLiP) model (Lobo, Arthur, & Laittimer, 2014). The CLiP model, developed in Amsterdam and adopted by Norfolk and Norwich University Hospitals in the UK National Health Service, uses the coaching method rather than traditional mentoring (Lobo et al., 2014). This model aims to challenge student knowledge, to encourage critical thinking and acquisition of skills, through the support of a staff nurse or practice educator (Taylor & Callow, 2017). In the model, students from all levels of study are allocated to the same clinical area, supported by the coach (Lobo et al., 2014). In some circumstances, the coach could also be another student who engages in shared learning with other students to support and learn from each other (Huggins, 2016).

A definition of coaching in nursing is a collaborative relationship between a coach and willing individual using conversation to help the individual achieve their goal (Donner & Wheeler, 2009). The role of the coach is not one of giving advice or teaching and does not offer direction. Instead, a coach acts to support and encourage the individual through the presented experiences (Donner & Wheeler, 2009). This differs from the ethos of PAL, giving evidence towards the shared learning and support of others to gain knowledge and skills (Topping, 2005).

In consideration of model for supporting learning, the UK Council of Deans of Health (2016) acknowledged PAL as an area for consideration when educating the nurses of the future to equip them to teach others, not just through mentorship, but also, informally, in the clinical environment. Considering these recommendations and the benefits of PAL it was clear that further synthesis of the available data was required to determine whether a conceptual model of PAL could be created. Therefore, this paper reports the outcomes of the meta‐ethnography undertaken to synthesize the available evidence exploring the influence of peer‐assisted learning on student nurses, when in clinical practice, to formalize a conceptual model for peer‐assisted learning.

## 2 METHODS

### 2.1 Design

A synthesis of the best available evidence was undertaken using meta‐ethnography to formalize a conceptual model for PAL. The meta‐ethnography used the findings of two observational studies, of which one was published (Carey, Chick, et al., 2018) and the findings of a systematic review of qualitative studies. The review used a detailed search strategy to identify and potentially include international literature; however, this found a lack of papers related to PAL in clinical practice (Carey, Kent, et al., 2018). An additional updated search in May 2018 for both UK and international papers did not find any new literature for inclusion. Research Ethics Committee Approval of the observational studies was obtained from the university ethics committee and the National Health Service (NHS, IRAS: 187233).

The synthesis of qualitative research is a concept that has been slowly gaining momentum in the area of health care (Dixon‐Woods, Agarwal, Jones, Young, & Sutton, 2005). However, challenges are presented in the form of choosing the best method for synthesizing qualitative studies. Terms such as meta‐analysis are often used to describe how quantitative research is synthesized. However, Barnet‐Page and Thomas (2009) noted that synthesis of qualitative data is more complex due to multiple associated terms and concepts that can be applied. One of these methods is meta‐ethnography, based on the influential work of Noblit and Hare (1988), to provide one alternative to meta‐analysis. The principle notion of this method fits with the aims of synthesis, whereby parts of research are brought together to construct a whole, which is greater than the sum of its parts (Barnet‐Page & Thomas, 2009).

The success of Noblit and Hare's synthesis was based on small groups, in two to six papers, of closely related studies, a similarity fitting in the context of the chosen larger project and its publications. However, the rationale for this choice of method compared with other examples of qualitative synthesis was the quality of the seven‐step process created for conducting meta‐ethnography (Noblit & Hare, 1988). Previous studies had evaluated the tool with claimed success in its ability to provide new conceptual development to build on existing theories (Britten, Campbell, Pope, Donovan, & Morgan, 2002; Campbell et al., 2003). The process also aided the development of models that interpret findings and reinterpret meaning across multiple studies (Atkins et al., 2008). Furthermore, the method was reported to be beneficial in identifying gaps that needed further research (Britten et al., 2002; Campbell et al., 2003).

Other considerations given to models for synthesizing qualitative research, included framework synthesis, derived from framework analysis (Ritchie & Spencer, 1994). This method offers a structured and rigorous approach to organizing and analysing large amounts of observations and field notes (Gail, Heath, Cameron, Rashid, & Redwood, 2013). The derivative of this is based on the same principle but applied across multiple studies (Barnet‐Page & Thomas, 2009). However, the basis of the synthesis for this project accounted for a smaller collection of chosen studies and a systematic review that had already gone through a comprehensive synthesis process using framework analysis (Carey, Chick, et al., 2018). This created a working framework, but also presented themes along with those as expressed in the systematic review, which were analysed using a qualitative data synthesis tool (Carey, Kent, et al., 2018).

### 2.2 Seven‐step process

The seven‐step process (Table 1) was used as the designated tool for conducting the secondary synthesis of a systematic review and two observational studies exploring PAL amongst undergraduate nurses in the fields of child health and adult nursing. Stage one (getting started) focuses on “finding something that is worthy of the synthesis effort” (Noblit & Hare, 1988, p. 27). As the topic was already the focus of the project this remained unchanged. Stage two implores the researcher to decide what is relevant to the initial interest, noting the process of conducting a systematic search, screening and appraising of all potential studies relevant to the synthesis. Following the implementation of a recent systematic review (Carey, Kent, et al., 2018), along with associated projects in the more recent observational studies, it was argued that all necessary studies had been identified for this stage.

**Table 1:** Noblit and Hare's (1988) seven‐step process for conducting meta‐ethnography

1. Getting started 2. Deciding what is relevant to the initial interest 3. Reading the studies 4. Determining how the studies are related 5. Translating the studies into one another 6. Synthesizing translations 7. Expressing the synthesis

Stage three encouraged reading and rereading the chosen studies ensuring that attention was paid to the details in the accounts (Noblit & Hare, 1988). This stage was helpful as it enabled the researchers to re‐familiarize themselves with the elements of the larger study, which led to stage four to determine how these studies are related to each other. Stage four identified and described metaphors, concepts or themes in each of the studies and compare them against each other. It was noted by Toye et al. (2013) that this process is fundamental to meta‐ethnography due to the concepts forming the raw data of the synthesis, but they highlighted the difficulties in deciphering concepts through their description. Other authors argued the challenges of interpretation to determine the distinction between findings and concepts when comparing data (Campbell et al., 2011). Toye et al. (2013), in their analysis of meta‐ethnography, discussed how these concepts can be broken down into an order of constructs. First‐order constructs depict the participant's interpretation in their own words, whereby second‐order constructs relate to interpretations of the researcher based on the data. First‐order constructs often only present a limited amount of original data, which in previous attempts has created difficulties to distinguish between first‐ and second‐order constructs and use these in the process (Smith & Anderson, 2018). Therefore, the aim of meta‐ethnography focuses on the data in second‐order constructs to synthesize and develop the researcher's interpretation of the original interpretations, or third‐order constructs (Britten et al., 2002). In this stage, second‐order concepts were considered to develop some initial interpretation.

As part of stage five, the presented key findings, which were made up of sub‐themes and key categories, were respectively chosen as second‐order constructs from the collective projects and translated according to interpretations by the primary researcher (Table 2). A recent study had adopted a similar approach to identifying and using key themes as their second‐order constructs to determine common and recurring themes (Strandås & Bondas, 2017). This stage involved exploring how these second‐order constructs were related through comparison. Toye et al. (2013) stated that constant comparing of these constructs enables the researcher to see similarities and differences between them and further create conceptual categories with shared meaning.

**Table 2:** Stage five, extract from translating studies into one another

Secondary construct	Concepts and translations
Complex choices when sharing learning opportunities	Undefined roles Role planning Organization
Delegation and planning roles	Clearly defined roles Role planning Organization
Discussion linked to best practice standards	Standards of practice Developing knowledge Learning Shared experience
Collaboration with nurse mentors	Confirming understanding Seeking guidance
Working together to provide patient care	Support in providing care Shared experience Developing skills Developing knowledge Learning
Increasing confidence/reducing anxiety	Decrease stress Reducing anxiety Enabling Improving self‐confidence
Supportive recognition of peers	Reducing anxiety Enabling
Connecting with peers to create bonding and mutual support	Friendships Social Naturally forming

**Figure 1:**
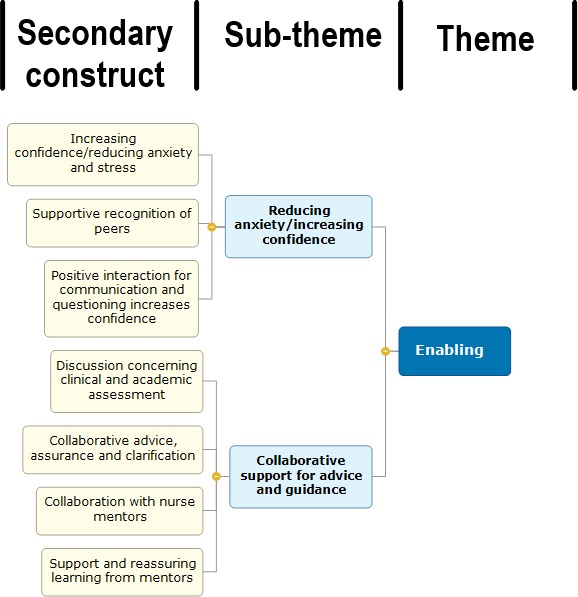
stage six, example of synthesizing translations for one theme

What follows next is stage six whereby the researcher uses interpretations of the secondary constructs to synthesize and make sense of the translation process. According to Noblit and Hare (1988), what should emerge is a set of three relationships where the analysis is either reciprocal, refutational or in line of argument. The synthesis process was conducted and presented as a flow diagram, an example of which can be seen in Figure 1. Previous studies had used similar examples for synthesizing translations, which offered a clear line of argument when undertaking this stage (Campbell et al., 2003; Purc‐Stephenson & Thrasher, 2010). The final stage in step seven concerns itself with the dissemination of the collected research and its findings to maximize impact (Toye et al., 2013). Once the translation of the synthesis had been completed, the primary researcher was able to adapt the flow diagrams into a conceptual model for PAL in clinical practice. To maintain credibility and trustworthiness, a review of each stage as well as any discrepancies were discussed with the research team. All themes and the final structure of the conceptual model were agreed by all authors.

## 3 RESULTS

**Figure 2:**
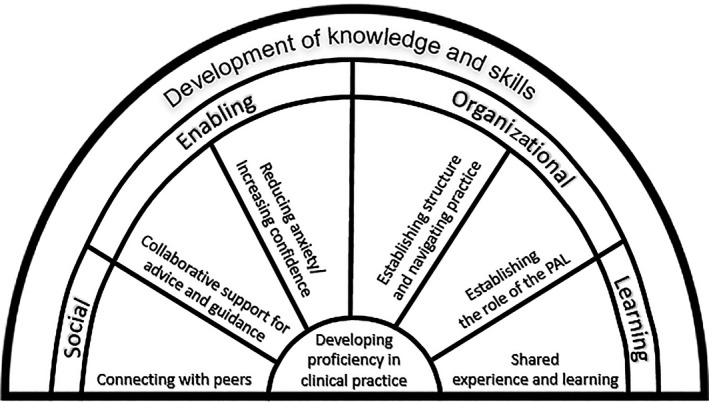
Conceptual model for peer‐assisted learning

The aggregated findings from secondary constructs were synthesized into four key themes; social, enabling, organizational and learning, underpinned by six sub‐themes that make up the conceptual model for PAL (Figure 2).

### 3.1 Social

The social theme is comprised of the sub‐theme: “connecting with peers,” where socialization forms an important part of PAL. Connecting with peers acknowledges how contributions towards PAL in practice are often underpinned by the communities formed by students who through shared status of equals develop friendships and practice socialization. The sub‐theme was aggregated from the secondary constructs; “connecting with peers to create bonding and mutual support” and “context for peer interaction and non‐interaction.” Connecting with peers to create bonding and mutual support takes into account how students converge together through their acknowledgement of their role, use each other as valuable sources of information and thus form their own community of support as captured in the systematic review:

Peers naturally form the friendships needed and practice socialization to use each other as resources. Through these actions, they develop their own specific communities.’…“the students see each other as a discrete group which only fellow students can understand and so develop their own parallel community to help each other: being in the same boat.” [Systematic review]

This sub‐theme also takes into consideration the context for peer interaction and non‐interaction, which further captures how peers interact with each other when engaging in PAL in clinical practice. Across both ethnographic studies exploring PAL amongst child health and adult student nurses, their interactions acknowledged socialization as a key part when engaging in PAL. This was particularly noted in the observational study with child health nursing students:

It was noted the context of how these interactions could be formed based on friendships and friendliness of other peers. The junior peer participants are all from the same year group and although they noted to have separate friendship groups, they gave a perceived impression of general acceptance and developing friendship towards one another. [Observational study nursing students: child health field]

### 3.2 Enabling

The enabling theme is comprised of two sub‐themes: “collaborative support for advice and guidance” and “reducing anxiety/increasing confidence.” The first sub‐theme, “collaborative support for advice and guidance” was aggregated from five secondary constructs: “discussion concerning clinical and academic assessment,” “positive interaction for communication and questioning,” “collaborative advice, assurance and clarification,” “collaboration with nurse mentors” and “support and reassuring learning from mentors.” This sub‐theme captures the importance of seeking advice, assurance and clarification for confirming understanding amongst student nurses engaging in PAL. In these situations, students practice their ability to seek support in PAL that enables them to move forward in developing their knowledge and skills when in clinical practice:

Questioning was highlighted as an approach to check understanding and seek clarification of a variety of clinical questions and tasks. Interestingly this offered reference towards perceived increased confidence through the availability of peers. [Observational study nursing students: adult field]

To ensure knowledge was correct, qualified nurse mentors were frequently called on as part of a process of clarification and confirmation of actions undertaken, generally when peer knowledge had reached its limit:

Collaboration between first year student nurses and one of the qualified nurse mentors to seek clarification on aspects of the observations they have taken. Mentor clarifies the accuracy of their query and encourages them to make this as a note on the observation chart. [Observational study nursing students: child health field]

The second sub‐theme, reducing anxiety/increasing confidence was aggregated from two secondary constructs; “Increased confidence/reducing anxiety and stress” and “Supportive recognition of peers.” This sub‐theme captures how students across both fields of nursing when engaging in PAL have increased confidence and are able to reduce anxiety through the support of their peer colleagues. Furthermore, as seen in previous extracts, the process of seeking clarification and advice has increased perceived confidence of peers through these actions.

Findings reflect how peer learning and support have perceived positive benefits in decreasing stress and anxiety and improving self‐confidence. [Systematic review]

### 3.3 Organizational

The organizational theme is comprised of two sub‐themes: “establishing structure and navigating practice” and “establishing the role of the PAL.” The first sub‐theme, “establishing structure and navigating practice” was aggregated from two secondary constructs: “peer support for infrastructure and environment” and “navigating the course in clinical practice.” Finding your way in practice was captured as a point of concern for student nurses, who often worked solo in their practice placement areas. Interestingly, students across the fields of child health and adult nursing found that engaging in PAL provided organizational benefits. Students were able to establish where they fit into the structure as student nurses and use each other for navigating clinical practice daily, using PAL as:

An opportunity to establish a better understanding of the working structure, but also where they fit into their role as student nurses. Interestingly some of the junior peers were located in different clinical areas, but the opportunity to support each other in navigation was not restricted to one particular clinical area. [Observational study nursing students: child health field]

The second sub‐theme, “establishing the role of the PAL” was aggregated from two secondary constructs; “complex choices when sharing learning opportunities” and “delegation and planning roles.” These constructs highlighted the importance of how student nurses establish their roles when engaging in PAL. The complex choices that students faced when engaging with PAL often came from challenges of negotiating tasks, personality clashes and more accurately student nurse peers lacking ability to clearly define their roles:

These arose occasionally in the area of accurately defining the roles of peers within the same pairs. This was evident in aspects of clinical task allocation. In these instances, it was noted by one peer that their partner was keen to take on the lion share of the clinical activity. [Systematic review]

Interestingly, the primary studies exploring the influence of PAL amongst groups of child health and adult student nurses found the reverse. In reports from these findings, it was noted how student nurses from both child health and adult fields took the time to establish their roles and responsibilities linked to task allocation:

The role of assigning a task or a role was presented as an open and fluid discussion between peers with an equal sharing of thoughts and ideas when engaging with PAL. Within these discussion peers were able to plan the task to be completed by defining the parameters and ensuring through effective communication that each person was clear on their role. [Observational study nursing students: adult field]

### 3.4 Learning

The learning theme is comprised of the sub‐theme: “shared experience and learning.” This sub‐theme takes into the account the opportunities for developing knowledge and skills and how PAL contributes towards students’ learning. The sub‐theme was derived from seven secondary constructs; “sharing of practice experience,” “enhancing knowledge of care,” “complementary learning aids clinical skill development,” “positive support linked to clinical observation and clinical task,” “informal interaction for teaching, learning and discussion,” “discussion linked to best practice standards” and “working together to provide patient care.” Viewing the concept of learning as a whole, student nurses who engage in PAL are able to develop their knowledge through discussing standards of care and their actions when working together as PALS. In addition, they were able to develop their skills as student nurses, helping to better apply these to their role and those under their care:

Together these findings reflect how peers work together to develop their clinical knowledge and skills, as well as their judgement to model effective care. [Systematic review]

Furthermore, learning was also obtained through student nurse peers who used shared experiences and previous knowledge to apply this in the opportunities presented when engaging in PAL:

The context for sharing of experiences were used to reinforce learning around topics of clinical practice. [Observational study nursing students: child health field]

## 4 DISCUSSION

This newly created conceptual model for PAL in clinical practice provides a structure where the overall goal is the development of knowledge and skills to help student nurses become more proficient in clinical practice. This follows a similar notion to the novice to expert model, whereby the acquisition of knowledge and skills is achieved through levels of proficiency, underpinned by experience and learning (Benner, 1984). Each of the four themes in the conceptual model highlights key elements required for student nurses to be able to develop the necessary knowledge and skills to enable them to become proficient as nurses in clinical practice.

Socialization practice appeared to be an important factor in peers connecting with each other to engage effectively in PAL when in clinical practice. The impact of socialization has been acknowledged as a key point of nurse education, whereby nurses need to be properly socialized in their profession to be effective in highly functioning teams (Levett‐Jones, Lathlean, Higgins, & McMillan, 2009; MacMillan, 2013). The role of modelling professional socialization has been highlighted as the responsibility of the qualified nurse mentor (Royal College of Nursing, 2017). Informal socialization between students and nursing staff has been acknowledged as a means to relieve stress; however, controversially students have also found themselves to be socially excluded by nursing staff (Levett‐Jones et al., 2009). In the engagement of PAL, students were able to develop community and a sense of belongingness for supporting one another in clinical practice. This finding was supported by Roberts (2009), who noted the importance of friendships amongst student nurses and encourage learning from one another.

One of the components that makes up part of the model was PAL being used for enabling the students to move forward in developing their knowledge and skills. This arose from the need for peers to be able to seek support of their colleagues through advice and clarification. A recent study captured the same in the support provided by student nurse peers in clinical simulation, whereby the ability to seek clarification and receive guidance from peers enabled knowledge and skills acquisition (Li, Petrini, & Stone, 2018). This was consistent in another study exploring peer facilitation in clinical skills, where support from peers enabled students to develop confidence in developing knowledge and skills to make them more proficient in their clinical practice (Davis & Richardson, 2017).

The concept of organization and establishing structure for students engaging in PAL is one that is missing from the current literature. Students, as they engage in PAL, can be used as a means to determine where they fit into the structure of clinical practice as student nurses. Aside from the larger project, one study noted the challenges of student nurses working solo, without the input of peers, as a barrier to determining where they fit into the working structure (Christiansen & Bell, 2010). Peer‐assisted learning appears to support development, particularly in relation to the knowledge of their student nurse role and thus enhance their ability to navigate the clinical area. Despite its support, it was clear that role allocation was a challenge for peers, who found it difficult to find the balance in sharing out clinical tasks. This could be a potential barrier to the organization of PAL in future research and therefore warrants further exploration.

The learning that is achieved when engaging in PAL, as outlined in theme four, is largely influenced by shared experience. When relating this back to Benner's (1984) Novice to Expert model, the importance of experience and its contribution towards proficiency in clinical practice is emphasized. Current standards for nurse education in the UK highlight the importance of proficiency as the end goal to enable students to qualify as nurses and progress onto the nursing register (Nursing and Midwifery Council, NMC, 2018). This is a task that is usually conducted by a registered nurse mentor; however, recent pressures on mentors, such as higher acuity of patients and staffing issues, make it more difficult to provide positive clinical learning experiences (Hanson, MacLeod, & Schiller, 2018). The changes about to be introduced by the NMC, following its review of standards for pre‐registration nursing (NMC, 2018) are designed to address such issues.

Peers working together were able to develop their knowledge and skills and reinforce learning through their own‐shared experience. This was directly observed by Austria, Baraki, and Doig (2013) who found that shared experiences amongst peers helped to fill in gaps in the students’ knowledge base. Furthermore, peers engaging in learning together helped each other to pass on practical skills through demonstrating and sharing through their own experiences (Roberts, 2008). This was often presented in senior to junior pairings when the sharing of knowledge and experience of senior students offered clear benefits to their junior colleagues (Austria et al., 2013; Carey, Kent, et al., 2018). However, it is important to acknowledge that shared knowledge and experience can benefit learning amongst student nurses in the same level of study. This has been captured in the experiences of practical skills presented by Roberts (2008) and with junior student nurses when assessing each other in their clinical skills (Rush, Firth, Burke, & Marks‐Maran, 2012). Considering the challenges faced by mentors and the support of learning provided by peers, it would be beneficial to consider whether the PAL model could provide a basis towards students assessing each other in the future alongside current models of mentorship.

### 4.1 Limitations

We acknowledge that the model was developed predominantly from a larger project and that not all elements were from published material. However, the available evidence of qualitative studies was included in the systematic review, and we also acknowledged that the exploration of PAL in nursing clinical practice is limited.

## 5 CONCLUSION

The importance of developing knowledge and skills to facilitate student nurses becoming proficient in clinical practice is a current point of focus in a changing healthcare workforce. The emphasis of educating the future nurse leans towards models of peer coaching, but does not discount the need to look at options, such as PAL. The aim of this study was to create a conceptual model of PAL, informed by a secondary meta‐ethnography that synthesized the evidence in a large study that considered the influence of PAL on student nurses in clinical practice. The development of this conceptual model presents a bold structure to outline the elements required for effective engagement and achievement of knowledge and skills attainment. These components highlight the previous captured data in the translation process and determine how these fit together to offer a more defined model that can be used in clinical practice as a structure for future engagement in PAL.

## 6 RECOMMENDATIONS FOR FUTURE RESEARCH

The next phase in the research process is to evaluate the influence of the conceptual model when implemented in clinical practice. This will determine how the model contributes towards the development of students’ knowledge and skills when engaging in PAL from the perspective of educators and the students themselves. The benefits of meta‐ethnography for identifying gaps in the research for further exploration were highlighted in the methods section (Britten et al., 2002, Campbell et al., 2003). On reflection of the newly constructed model, it is clear that the organizational principle presented data that polarized student perceptions in establishing the role of the PAL. It is therefore recommended that further research would be required to explore how student nurses establish their roles when engaging in PAL in clinical practice. Further research is also required to consider the transition of student nurses with previous healthcare experience and how their support might differ when moving into a new role.

## CONFLICT OF INTEREST

The corresponding author and co‐authors confirm that there are no conflicts of interest.
